# Predicting the Phenology of Herbivorous Insects

**DOI:** 10.1002/ece3.71734

**Published:** 2025-07-14

**Authors:** Zimo Yang, Elise Woodruff, David Held, Nate B. Hardy

**Affiliations:** ^1^ Department of Entomology and Plant Pathology Auburn University Auburn Alabama USA

**Keywords:** degree days, integrative pest management, lower developmental threshold, multilevel Bayesian models, phylogenetic models, phytophagy, thermal requirements

## Abstract

Models of herbivorous insect phenology can be used to make agriculture more sustainable and to better manage the effects of climate change on natural communities. The phenology of herbivorous insects depends on heat time, but exactly how it varies across populations and the causes of this variation are unclear. Here, with multilevel Bayesian models, we performed a comparative analysis of 601 published herbivorous insect phenology models. We found that variation in herbivorous insect phenology can be explained by variation in phylogenetic relatedness, adult body size, feeding site, host plant taxonomy, geographic location, and the approaches that researchers used for model parameterization. Contrary to previous analysis, we also found that the minimum temperature required for development varies across life stages in a way that could be adaptive. Our analysis demonstrates that by accounting for more information on the variation across insect populations and their environments, we can make better and more generalizable predictions of herbivorous insect phenology.

## Graphical abstract

In this article, we investigate factors, other than temperature, that influence herbivorous insect phenology. We compare published herbivorous insect phenology models parameterizations with multilevel Bayesian models. We find that (1) variation in herbivorous insect phenology can be explained by variation in insect taxonomy, adult body size, feeding site, host plant taxonomy, geographic location, and how a model is parameterized, and (2) the minimum temperature required for development tends to vary across developmental stages.

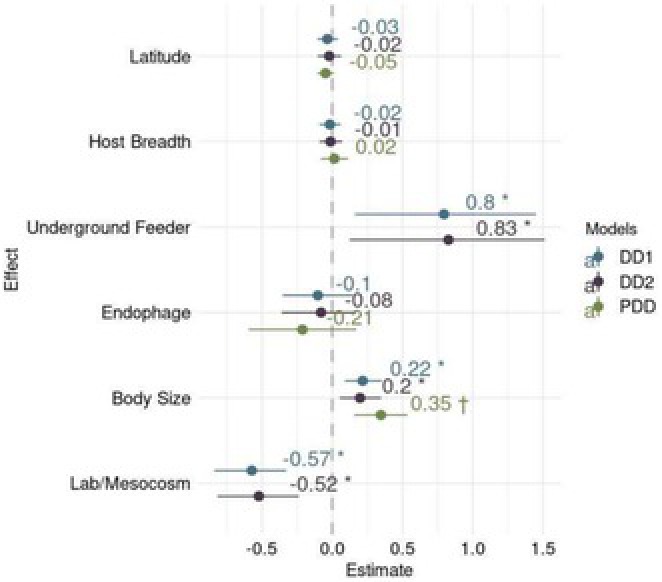

## 1 Introduction

Heat governs development in poikilothermic organisms, and temperature‐based phenology models have long been used to predict the timing of insect development, with the aim of promoting more precise and sustainable integrative pest management (Welch et al. 1978). The importance of phenology models for pest management is likely to increase as climate change accelerates development and shortens susceptible phenophases in pest populations (Gagnon et al. 2019). Phenology models also have many other general applications related to climate change. For example, they can help us anticipate changes in species distributions (Chuine 2010), interactions (Matthysen et al. 2011; Renner and Zohner 2019; Schneider et al. 2022; Tylianakis et al. 2008), and consequent changes in community structure and function (Krinner et al. 2005; Piao et al. 2019; Suttle et al. 2007; Yang and Rudolf 2010). Nevertheless, as it stands, the usefulness of temperature‐based phenology models is rather limited to the populations from which models were parameterized.

Indeed, mapping of heat time to phenology varies considerably across populations (e.g., Bentancourt et al. 1996; de Campos et al. 2021; Mohamed et al. 2022; Özgökçe et al. 2016). Although many hundreds of population‐specific models have been parameterized, we have a poor understanding of what causes this variation. Consequently, we are unable to generalize from what we know about the phenology of some populations to the phenology of the many populations which have yet to be characterized. Here, our goal was to make phenology models more predictive and generalizable for herbivorous insects, a group for which phenology modeling can be especially practical. Our approach is to compare the published models.

## 2 Materials and Methods

### 2.1 Data

The classical approach is to explain insect phenology as a simple, usually linear, function of accumulated heat time: *D* = *DD*/(*T*‐*LT*), where *D* is the length of a developmental stage (typically measured in days), *T* is temperature (°C), *LT* is the lower threshold temperature for development (°C), and *DD* is the population‐specific heat time needed to complete a developmental stage (°C*days = degree days). More recently, more complex, nonlinear functions have been used, but these are approximately linear over much of the typical range of temperatures that insect populations would experience (Rebaudo and Rabhi 2018). For the sake of comparative breadth, we focused on linear models. To parameterize such a model, one need only estimate values for *DD* and *LT*. Two main estimation methods have been used. The first entails experimental measures of developmental time over a range of constant temperatures. In that case, *LT* and *DD* can be estimated from a regression of daily development rate (*D* ^−1^) against *T* (Arnold 1959; Campbell et al. 1974; Logan et al. 1976). The second approach entails field‐based observations of development time and heat time, in which case some kind of average temperature per day is used for *T* (Rodríguez Caicedo et al. 2012). Studies of this kind tend to recycle values for *LT* derived from controlled experimental approaches (e.g., Whitford and Quisenberry 1990).

Our comparative dataset builds on the Database of Thermal Requirements (DTR) of Jarošík et al. (2011), which is itself an extension of the Insect Development Database of Nietschke et al. (2007). The DTR covers more than 500 insect species, 310 of which are herbivorous, with data from 1034 studies published between 1972 and 2004. Each record in the database gives estimates of *LT* and *DD* for development from egg to adult and for component life stages (e.g., the pupal stage), along with meta‐data about the model's publication and the focal insect's taxonomy and study location. We extended the herbivorous insect part of the DTR in two ways. First, we added more recently published phenology models. Second, we included several potentially explanatory predictors of phenological variation.

To find phenology models published after 2004, we conducted a systematic literature search. This comprised two queries of Google Scholar, the first with the search terms “insect” AND “phenology model” AND “temperature,” and the second with the terms “insect” AND “model” AND “temperature‐dependent” AND “develop*.” We analyzed the first 1000 matches returned by each query. The relevance of each publication was assessed by scanning the title, abstract, methods, and results sections. Our criteria for inclusion were that (1) a study reported at least one measure, based on an explicit model of empirical data, of the dependency of a nondormant herbivorous insect population's phenology on temperature, or (2) such a quantity could be derived from the reported phenology model. A total of 219 studies met these criteria and provided an additional 301 records to the dataset. On the other hand, application of these same inclusion criteria to the original data in the DTR of Jarošík et al. (2011) resulted in the exclusion of 10 records that lacked an explicit empirical basis. In total, we obtained 601 herbivorous insect phenology model records.

In addition to removing certain data from the original DTR, we amended the DTR data in three ways. (1) In some cases, the phenology model parameter values in the original DTR deviate from values in the original studies, as they were recalculated to only include life table data under temperatures for which development is actually linear (Jarošík et al. 2011). For the sake of homogeneity across models, we reverted these records back to the originally reported values since there was no truncation of experimental temperatures for models that were originally presented as linear. (2) Assuming that *LT* is constant across life stages (Jarošík et al. 2002), in some cases Jarošík et al. (2011) approximate *LT* for total development from egg to adult with the *LT* estimated for any component life stage (i.e., the egg stage, the growth stages, or the pupa). Here, we did not assume that *LT* is fixed across life stages and instead focused on life‐stage component estimates rather than whole life‐cycle approximations. (3) Jarošík et al. (2011) record estimates of *DD* for total development from egg to adult only when such a value is reported by the primary model authors. In contrast, in 139 cases, we approximated *DD* for total development as the sum of *DD* values for component life stages. To check that this approach was reasonable, from 106 records in which total development time is reported alongside development time for each component life stage, we calculated the proportional difference between the reported total and our approximation. Such differences were negligible; they were normally distributed with a mean of 1.04% and a standard deviation of 7.70% (Shapiro–Wilk normality test: *p* value < 0.01).

We defined *LT* strictly as the temperature under which no development occurs and defined *DD* as the heat time above *LT* required to complete a phenophase in a controlled experiment, or to reach 50% of cumulative capture in the field. When a study models multiple nondormant generations, we took the average value across generations.

The variables we used to explain phenological variation are summarized in Table 1. This includes five fixed effects: (1) *Measurement type*. As previously mentioned, researchers have parameterized herbivorous insect phenology models either via laboratory experiments or field‐based observations. Here we included measurement type as a binary variable with the states “laboratory/mesocosm” and “field.” (2) *Latitude*. One might expect that to get the most out of relatively short growing seasons, herbivorous insect populations at higher latitudes will evolve lower values for *LT*. To include an estimate of the latitude at which each population was sampled, we geocoded (Free Geocoding API, n.d.) the provenance data provided in the TRD or by the authors of more recent phenology models. When phenology models were parameterized from a combination of source populations, we used mean geocoded latitude. Estimates of latitude were dropped for any model for which the provenance data were sufficiently imprecise or broad that the span of possible latitudes was greater than 10°. (3) *Diet breadth*. Although the empirical evidence is equivocal, host‐use specificity could affect the conversion efficiency of feeding (Hardy et al. 2020). Therefore, we included an estimate of the diet breadth of each herbivorous insect species, based on a count of documented host–plant species (data sources are provided in Zenodo: DOI: 10.5281/zenodo.15792706). (4) *Feeding site*. Some insects feed on more insulated parts of their host plants than others, and thus may be less sensitive to changes in the flux of heat time. Therefore, each population was classified as either (i) an exposed aerial feeder, that is, exophagous, (ii) a concealed aerial feeder, that is, endophagous, for example a leaf miner or gall inducer, or (iii) an underground feeder. (5) *Body size*. One of the most obvious variables that could affect phenology is body size; some insects have more growing to do than others. In the database (DOI: 10.5281/zenodo.15792706), body size is most often expressed simply as an estimate of average body length, measured from the anterior end of the head to the posterior end of the abdomen. However, for some taxa (e.g., butterflies), body size is more often expressed as a measure of wingspan or forewing length, and in fact, measurements of body length are hard to come by. Although we included a mix of body size indices in the database, we only analyzed records in which it is measured as the length from head to tail.

**TABLE 1:** Potential predictors of the variation in herbivorous insect phenology model parameters.

Variable	Description	Variable type	Variable states	Potential relevance
Host‐use breadth	Number of known host plant species	Fixed effect	Numeric; log‐transformed	Feeding conversion efficiency; synchronization with host phenology
Feeding site	Trophic mode during growth stages	Fixed effect	Discrete: exposed aerial tissues, conceal areal tissues, underground tissues	Some host tissues more insulated from fluxes of heat
Adult body size	Length in millimeters	Fixed effect	Numeric; log‐transformed	Larger bodies take more development
Source population latitude	Central latitude of insect sampling location in decimal degrees	Fixed effect	Numeric; absolute value	Captures many unmeasured environmental variables
Measurement type	How an insect phenology model was parameterized	Fixed effect	Discrete: Lab/mesocosm, field experiment	Measurement bias
Host plant taxonomy	Classification of experimental host plant	Random effect	(a) Family (b) Nesting of family: genus	Phylogenetic conservatism of host plant features
Insect taxonomy	Classification of herbivorous insect	Random effect	Nesting of order: family	Phylogenetic conservatism of insect features
Insect phylogeny	Evolutionary relationships among species, with a branch length unit of 1 million years	Random effect	Variance–covariance matrix	Phylogenetic conservatism of insect features

Our models of phenological variation also include three kinds of random effects: (1) *Insect co‐ancestry*. We accounted for shared ancestry in two ways. In what we refer to as taxonomy models, we included the order‐ and family‐level classification of each insect species as nested random effects. We did not include random effects for lower taxonomic levels to avoid over‐parametrization; too few phenology models were available for most taxa below the family level. For a complementary accounting of the effects of insect co‐ancestry, in what we refer to as phylogeny models, we included a phylogenetically derived variance–covariance matrix as a random effect. For the phylogeny, we used the relationships among 217 of the 419 species in our database that are represented in the “TimeTree” (Hedges et al. 2015; Kumar et al. 2022). Species not found in the TimeTree were dropped from the phylogeny models. To include the multiple phenology models available for some species, we replaced certain tip nodes (i.e., species) in the TimeTree phylogeny with as many random bifurcations as there were models for that species, giving a trivial branch length (1*e* ^−6^ million years of divergence) to each new “pseudotip” and, to keep the phylogeny ultrametric, subtracting an equal amount from the branch subtending the pseudotip clade (Figure S2). From this amended TimeTree phylogeny, we obtained a variance–covariance matrix by assuming that features in the database evolved via Brownian model adjusted by Pagel's *λ* (Freckleton et al. 2002; Pagel 1999), using the *corPagel* function of the R package *ape* (Paradis et al. 2024). We estimated the most likely *λ* value by fitting a series of models assuming *λ* values between 0 and 1, with an increment of 0.01, and then selecting for the *λ* value with the best model AIC score. (2) *Diet*. Previous research has demonstrated that changes in host use can modify the mapping of heat time to phenology in herbivorous insects (Clancy and Price 1987; Feeny 1976). Therefore, we characterized the host usage of each modeled insect population with the family‐ and genus‐level classification of their host plant. In some experimental parameterizations of phenology, insects were fed an artificial diet. This was included as an additional level in the family classification, with the corresponding genus‐level state left empty. In some field‐based parameterizations of polyphagous populations, the diet status is uncertain, and for these records in the database, the diet variable was left unspecified. (3) *Measurement type*. In phylogeny models, we included measurement type as a random variable instead of a fixed variable, since it captures variation in how researchers have tried to observe population features, rather than variation in those features themselves, and it would be inappropriate to use phylogenetic models for such observational variation. Measurement type was quantified in the same way, laboratory‐based versus field‐based, regardless of whether it was treated as a fixed or random effect.

### 2.2 Analysis

Phenology models missing data for a variable included in one of our comparative models were excluded from the analysis. We performed all analyses in R 4.4.2 (R Core Team 2024). To increase normality, *DD*, insect host‐use breadth, and insect body size were log‐transformed. Insect host‐use breadth, insect body size, and latitude were standardized by means in units of standard deviations. To address our main question about what affects variation in herbivorous insect phenology models across populations, we fit three groups of models: One group with the *LT* for the egg stage as the response, a second with the *LT* for growth stages as the response, and a third with *DD* as the response. For each of these groups, we fit a model variant for each of three different random effect structures: (i) An insect taxonomy model including only the family‐level host plant taxon as a random effect, and with artificial diet as an extra level in that classification, (ii) an insect taxonomy model with nested random effects for family‐ and genus‐level host plant classification that excluded insect populations fed on artificial diet, and (iii) an insect phylogeny model with family‐level host taxon as a random effect.

Additional taxonomy models were fit to assess the variation of *LT* across life stages. These models use all of the same fixed and random effects found in the main models, but *LT* is the response variable, life stage is a fixed effect with three levels: egg, growth stages, pupae, and an additional “study” random effect is included to account for repeated measures from individual experiments. Previous work suggests that *LT* may be fixed across life stages (Jarošík et al. 2002). But *LT* differences across life stages could be involved in the adaptive timing of development. One hypothesis is that the egg *LT* tends to be lower than that of subsequent life stages, simply because eggs come first, when environments tend to be colder, and a lower *LT* for eggs would allow for growth to begin earlier in the season. On the other hand, a higher *LT* for eggs could also be adaptive, either by facilitating phenological synchronization with host plants or by reducing the risk of exposure to bouts of extreme cold in early spring, with the added notion that episodes of stalled metabolism might increase vulnerability to predation.

We fit all taxonomic models with Bayesian method with *R* function *brm* from the *brms* package (Bürkner 2017). We used uninformative priors for each parameter. Searches used four parallel chains of 4000 iterations, with a 2000‐iteration burn‐in, a thinning rate of one iteration, and a target average proposal acceptance probability of 0.95. We checked the goodness of fit of each model with *R* ^2^ values with *R* function *bayes_R2* from the *brms* package (Bürkner 2017). Bayesian inferences on phylogeny models did not converge well, so we fit those models with Maximum Likelihood using the *R* function *pglmm* from the *phyr* package (Ives et al. 2020). We obtained *R* ^2^ for each phylogeny model with *R* function *R2_lik* from the *rr2* package (Ives and Li 2023).

Model parameters are summarized in Table 2. The updated herbivorous insect phenology model database and codes to reproduce the models are available as a Zenodo repository (https://zenodo.org/records/15792706).

**TABLE 2:** Model variants. We built multiple sets of models with the same respond variables to test the sensitivity to model design. We handled experiments involving artificial diets in two ways: (a) in one set of models, they were included as a level in the family classification of plant hosts, with only that taxonomic level included as a random effect; (b) in other set of models, they were excluded and we used nested random effects for plant families and genera. In most models we accounted for insect relatedness with nested random taxonomic effects. For a subset of species, we also fit phylogenetic mixed models.

Model name	Respond variable	Fixed effects	Random effects
LTE1	*LT* for egg	All effects in Table 1	Insect taxonomy + host taxonomy (a)
LTE2			Insect taxonomy + host taxonomy (b)
LTL1	*LT* for growth stages^a^		Insect taxonomy + host taxonomy (a)
LTL2			Insect taxonomy + host taxonomy (b)
DD1	*DD* from egg to adult		Insect taxonomy + host taxonomy (a)
DD2			Insect taxonomy + host taxonomy (b)
PLTE	*LT* for egg	All effects in Table 1 except measurement type	Insect phylogenetic variance–covariance matrix + host taxonomy (a) + measurement type
PLTL	*LT* for growth stages		
PDD	*DD* for egg to adult		
LTstage1	*LT* for egg, growth stages, and pupae	All effects in Table 1 + developmental stage	Insect taxonomy + host taxonomy (a) + experiment
LTstage2			Insect taxonomy + host taxonomy (b) + experiment

^a^ Here we use the general term “growth stages” to refer to all immature stages following the egg and before the pupa in holometabolous insects or the adult in nonholometabolous insects.

## 3 Results

### 3.1 Database Overview

The expanded herbivorous insect phenology model database comprises 607 models. The taxonomic coverage roughly corresponds to the species richness and economic importance of herbivorous insect clades (Table S1). After filtering out experiments with excessively imprecise provenance data (latitudinal range > 10°), the Northern hemisphere was over‐represented in comparison with the Southern hemisphere (df: 1, *χ* ^*2*^: 144.22, *p* value < 0.01), while tropical (< 23.5° NS) and cold zones (> 60° NS) were under‐represented in comparison with subtropical and temperate zones (< 23.5°, < 60° NS) (df: 2, *χ* ^*2*^: 144.22, *p* value < 0.01, Figure S1).

### 3.2 Taxonomy Models

In taxonomy models of the variation in *LT* and *DD*, both the fixed and random effects were important predictors (Table 3). Random model intercepts for each insect and host taxon are provided in Table S2–S7. Across models, insect taxonomy explained more phenological variance than diet taxonomy, regardless of how the latter was expressed (Table S8). Due to low sample size, we could not reliably estimate effects for each specific insect taxon (Figure S3). Below, as we report specific effect estimates, we use the notation introduced in Table 2 to refer to specific model variants.

**TABLE 3:** *R* ^2^ values and sample sizes. See Table 2 for model design. PLTE, PLTL, PDD are phylogenetic maximum likelihood models for which we were unable to calculate a marginal *R* ^2^.

Model	Sample size	Conditional *R* ^2^	Marginal *R* ^2^
LTE1	123	0.57	0.16
LTE2	102	0.60	0.15
LTL1	138	0.58	0.17
LTL2	115	0.65	0.16
DD1	166	0.62	0.25
DD2	143	0.66	0.23
PLTE	50	0.48	—
PLTL	63	0.62	—
PDD	71	0.79	—
LTstage2	251	0.69	0.18

**FIGURE 1:**
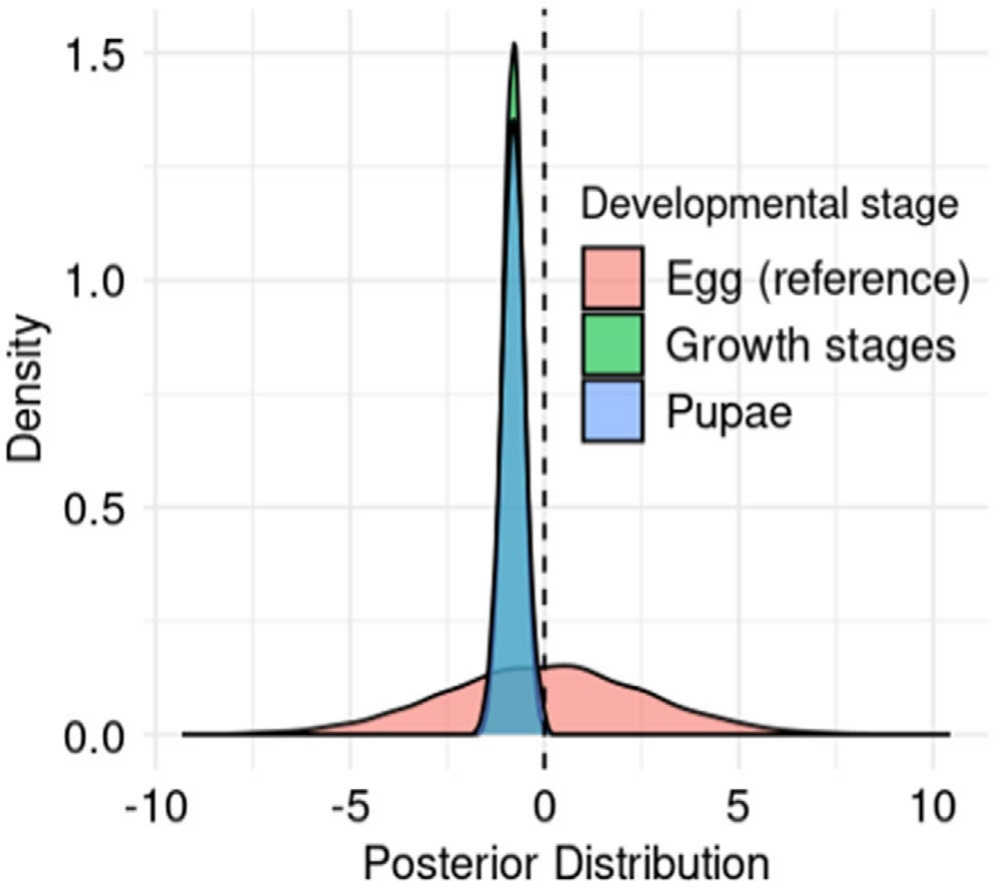
Variation across life stages in *LT*, the lower temperature threshold for development. The plot is based on model LTstage2 (see Table 2). Egg stage is the reference level and its posterior distribution was standardized by subtracting the mean value. The dashed line marks the mean egg‐stage effect. *LT* is significantly lower for growth stages and pupae than for egg, but does not significantly differ between growth stages and pupae (see Section 3).

**FIGURE 2:**
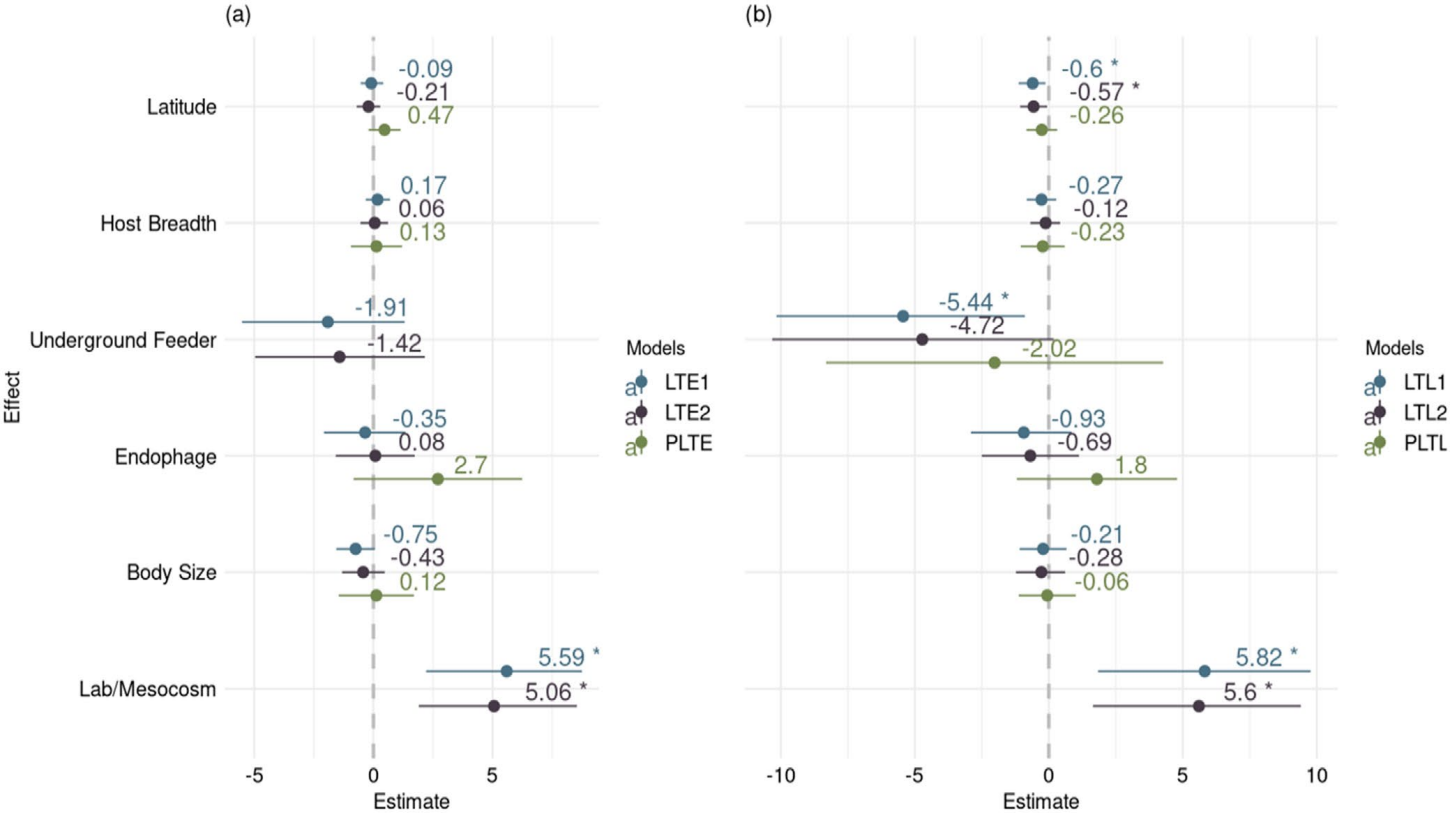
Fixed effects on *LT*, the lower threshold for development, for the (a) egg and (b) growth stages. *LT* was measured in °C. Host breadth and body size were log‐transformed. Latitude, host breadth, and body size were centered on the mean and scaled by standard deviations. Error bars represent 95% high density intervals (HDI) for taxonomy models and 95% confidence interval (CI) for phylogenetic models. Effects for taxonomic models with the experimental host–plant family as a random effect (LTE1 and LTL1) are shown in blue, while those included nested effects for host–plant family and genus (LTE2 and LTL2) are in violet. Phylogenetic model effects are shown in green. Effect estimates with a 95% HDI or CI that does not span zero are marked with an asterisk. No effects in the phylogenetic models (PLTE and PLTL) met that criterion. The reference level for insect feeding site is “exophagous.” The reference level for measurement type is “field experiment.” The phylogenetic models do not include measurement type. Note that the phylogenetic model of the egg *LT* (PLTE) does not include any example of underground feeders.

**FIGURE 3:**
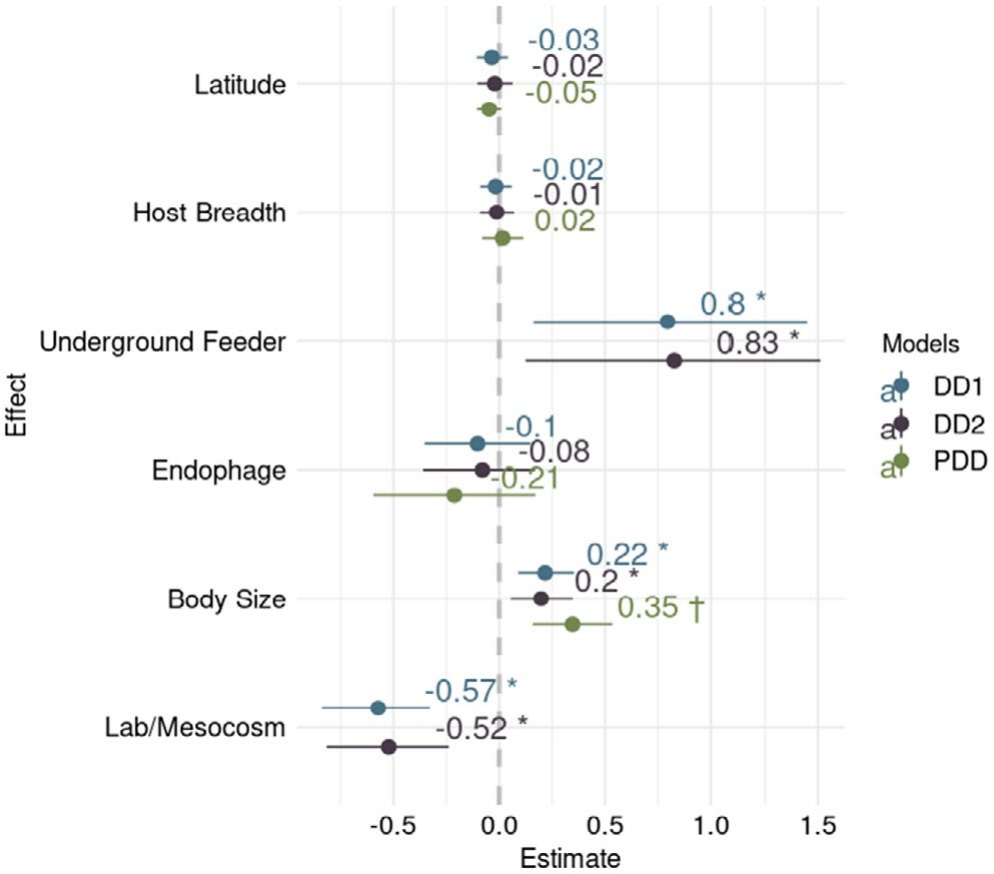
Fixed effects on *DD*, the heat time required for development from egg to adult. *DD* was log‐transformed and measured in degree days. Model names are taken from Table 2. † indicates that the effect has a *p* value < 0.05 in phylogenetic models. Error bars represent 95% high density intervals (HDI) for taxonomy models and 95% confidence interval (CI) for phylogenetic models. Effects for the taxonomic model with the experimental host–plant family as a random effect (DD1) are shown in blue, while those including nested effects for host–plant family and genus (DD2) are in violet. Phylogenetic model (PDD) effects are shown in green. Effect estimates with a 95% HDI or CI that does not span zero are marked with an asterisk. The reference level for insect feeding site is “exophagous.” The reference level for measurement type is “field experiment.”

For *LT*, by and large, our fixed effect inferences are consistent across model variants (Figures 2 and 3). How a phenology model is parameterized has a large effect on the *LT* (Figure 1); in comparison to field‐based parameterizations, laboratory‐based parameterizations tend to estimate significantly higher values for egg *LT* (LTE1, estimate: 5.59, 95% HDI: [2.22, 8.75]; LTE2, estimate: 5.06, 95% HDI: [1.90, 8.54]) as well as the growth stages *LT* (LTL1, estimate: 5.82, 95% HDI: [1.84, 9.77]; LTL2, estimate: 5.60, 95% HDI: [1.65, 9.41]). As expected, insects sourced from higher latitudes tend to have a lower *LT* for the growth stages (LTL1, estimate: −0.60, 95% HDI: [−1.13, −0.12]; LTL2, estimate: −0.57, 95% HDI: [−1.07, −0.06]), but not for the egg. Also, with the family‐level classification of diet, including artificial ones, as a random effect, *LT* for growth stages tends to be lower in species feeding on underground plant tissues than exophagous species (LTL1, estimate: −5.44, 95% HDI: [−10.16, −0.89]; post hoc test, estimate: 5.46, 95% HDI: [−10.16, 0.90]) and endophagous species (LTL1 post hoc test, estimate: 4.51, 95% HDI: [−9.02, −0.15]). Finally, *LT* is not significantly affected by diet breadth.

For *DD*, we also found that laboratory‐based parameterizations tend to indicate that less heat time is required to complete development (DD1, estimate: −0.57, 95% HDI: [−0.84, −0.32]; DD2, estimate: −0.52, 95% HDI: [−0.82, −0.24]). We also found that values of *DD* are positively correlated with body size (DD1, estimate: 0.22, 95% HDI: [0.09, 0.35]; DD2, estimate: 0.20, 95% HDI: [0.05, 0.35]). Furthermore, species feeding on underground plant tissues tend to require more *DD* than exophagous species (DD1, estimate: 0.80, 95% HDI: [0.16, 1.45]; DD2, estimate: 0.83, 95% HDI: [0.12, 1.51]; post hoc tests give the same estimate and HDI) and endophagous species (DD1 post hoc test, estimate: 0.90, 95% HDI: [0.26, 1.54]; DD2 post hoc test, estimate: 0.9, 95% HDI: [0.20, 1.58]). In contrast, we recovered insignificant effects from latitude and diet breadth.

### 3.3 Phylogeny Models

We found evidence of a strong phylogenetic signal for the *LT* of the egg stage (Pagel's *λ* = 0.86), *LT* for growth stages (Pagel's *λ* = 0.86), and *DD* (Pagel's *λ* = 0.98). However, because many species were not represented in our phylogeny estimate, the phylogeny model inferences were based on fewer samples than the taxonomy models, no field‐based records were present in the analysis of egg *LT*, and no underground‐feeding species were represented in the models of *DD* and egg *LT*. The egg *LT* model also has a relatively low goodness of fit (Table 3). Nevertheless, the effect of phylogeny per se was important (Table S8), and barring a few exceptions, the phylogeny model inferences are consistent with those from taxonomy models (Figures 2 and 3). Exceptions are that in the phylogeny models, the *LT* for growth stages is not significantly affected by latitude (PLTL, estimate: −0.34, 95% CI: [−0.95, 0.28], *p* value: 0.28) or feeding on underground plant tissues (PLTL, estimate: −1.98, 95% CI: [−8.50, 4.55], *p* value: 0.55). As a random effect, variation in model measurement type still explained a great part of the variance in both *LT* and *DD*, corresponding to its significant and strong effects as a fixed effect in taxonomy models.

### 3.4 Variation in LT Across Life Stages

Bayesian model estimates converged only for the model variant including nested effects from the host plant genus and family classification, and excluding experiments that used an artificial diet. In contrast to previous analysis (Jarošík et al. 2002), we found that *LT* tends to be significantly lower for growth stages than for the eggs (LTstage2, estimate: −0.81, 95% HDI: [−1.32, −0.31]; post hoc test, estimate: −0.80, 95% HDI: [−1.43, −0.24]). We also found a significant reduction in *LT* from egg to pupa (LTstage2, estimate: −0.80, 95% HDI: [−1.43, −0.24]; post hoc test, estimate: −0.80, 95% HDI: [−1.32, −0.31]), while *LT* does not differ significantly between growth stages and pupae.

## 4 Discussion

Before this study, insect phenology was known to vary systematically across major insect taxa, but more specific organismal and environmental features underlying this taxonomic effect had not been determined (Jarošík et al. 2011; Nietschke et al. 2007). Here, for herbivorous insects, we recover significant effects from body size, feeding site, and latitude. It seems that herbivorous insect development tends to take longer when the adult form is larger (D'Amico et al. 2001; Davidowitz et al. 2003) and when they feed in more insulated microenvironments.

Likewise, the minimum temperature required for development to progress through the growth stages tends to be lower at higher latitudes, where growing seasons tend to be shorter. On the other hand, diet breadth would seem to be less consequential, which can be taken as evidence against the oft‐assumed negative correlation between diet breadth and feeding efficiency (Hardy et al. 2020).

The most important predictor of phenological variation proved to be how a phenology model is parameterized. Compared with phenology models based on field observations, laboratory‐based experiments consistently estimate higher *LT* and lower *DD* values. On one hand, when herbivorous insects are in the field, cues of predation risk can alter foraging behavior and reduce foraging efficiency (Danner and Joern 2003; Sih 1980). On the other hand, to varying degrees, in the field, herbivorous insects can choose their host plants to maximize their feeding efficiency (Behmer and Joern 2008; Clissold and Simpson 2015). Thus, systemically higher estimates of *DD* from the field could indicate that the inhibitory indirect effect of predators tends to outweigh the positive effects of host choice. Furthermore, whereas most experiments entail constant temperatures, in the field, insects are exposed to natural temperature fluctuations. Such fluctuations could allow insects to start development when daily mean temperature is lower than their *LT* (Colinet et al. 2015). Moreover, insects in the field may also have a higher capacity for behavioral thermoregulation, for example, by basking. Field studies can therefore lead to lower *LT* estimations. That being said, temporal variation in temperature could have more complex effects on insect phenology; this is an open research question (Colinet et al. 2015).

Our analysis recovered strong random effects from insect co‐ancestry on *LT* and *DD*, but we lacked sufficient comparative data to characterize such effects at the level of specific insect taxa. That being said, the fact that we recovered a high phylogenetic signal for phenology model parameters suggests that the phenological variation within species pales in comparison with that between species and taxa of higher rank.

Our analysis rejects the hypotheses that *LT* is fixed across insect life stages (Jarošík et al. 2002). Instead, we found that the egg *LT* is generally higher than for subsequent development stages (Figure 1). A higher *LT* for eggs could be adaptive in various ways, for example, by facilitating phenological synchronization with host plants or by reducing the risk of exposure to bouts of extreme cold in early spring. In any case, heterogeneity in *LT* across life stages suggests that heat time both fuels insect development and provides information that insects use to time development.

Our inferences are contingent on certain caveats. First, the source populations of the phenology models in our analysis are biased toward the mid‐latitudes of the Northern Hemisphere. We are unaware of any reason why such geographic bias would invalidate our attempts to explain and generalize from the variation across insect taxa in *LT* and *DD*. On the other hand, such a bias could affect our inference of variation in LT across life stages, as we suspect that such variation could be an adaptive response to environmental uncertainty in temperate regions. Furthermore, although herbivorous insect phenology is almost certainly influenced by abiotic variables other than temperature, our characterizations of that variation are quite coarse; we relied heavily on a rough approximation of insect source population latitude. Future efforts to model insect phylogeny as a more complex function of multiple climate variables, especially variation in precipitation, may be especially fruitful. Also, phenological variation across populations is certain to have a population genetic component that is entirely missing from our comparative analysis (although we do account for deeper phylogenetic constraints). Along with a richer account of climate variation, a better understanding of the genetic basis of insect phenology variation could usher in a new era of more powerfully predictive models. Nevertheless, our multivariate mixed models provide an explicit framework for generalizing our knowledge of herbivorous insect phenology and can help us make better phenology predictions.

## 5 Conclusion

The context dependence of insect phenology has spurred the development of myriad population‐specific phenology models, but the lack of a general framework for insect phenological variation strongly limits the scope of applicability of the phenological theory, a problem that is becoming ever more acute as biotas mix and climates and landscapes change more rapidly. By comparing hundreds of published phenology models developed for specific herbivorous insect populations, we found several variables that can be used to explain phenological variation: insect taxonomy, host taxonomy, body size, feeding site, and latitude. We also uncovered evidence of strong systemic biases in phenology characterizations stemming from how they are characterized. We found that contrary to previous work, the minimum temperature required for herbivorous insect development varies significantly across life stages. We see these inferences as contributing toward a nascent general theory of insect phenology and look forward to the day when that theory can be refined via analysis of richer descriptions of how phenology varies across insect genotypes and environments.

## Author Contributions

**Zimo Yang:** conceptualization (supporting), data curation (lead), formal analysis (equal), investigation (equal), methodology (equal), software (equal), visualization (lead), writing – original draft (lead), writing – review and editing (supporting). **Elise Woodruff:** conceptualization (supporting), data curation (supporting), investigation (equal), methodology (equal), software (equal). **David Held:** conceptualization (lead), funding acquisition (lead), investigation (supporting), project administration (equal), supervision (equal), writing – review and editing (lead). **Nate B. Hardy:** conceptualization (equal), data curation (supporting), formal analysis (equal), funding acquisition (equal), investigation (equal), methodology (equal), project administration (equal), software (equal), supervision (equal), visualization (supporting), writing – review and editing (lead).

## Conflicts of Interest

The authors declare no conflicts of interest.

## Supporting information


Data S1.

## Data Availability

Data and codes to reproduce models are in Zenodo: https://zenodo.org/records/15792706.
