# Supplementary material for: Predicting the Phenology of Herbivorous Insects
Source: Ecol Evol. 2025 Jul 14;15(7):e71734. doi: 10.1002/ece3.71734 (PMC12256772; doi:10.1002/ece3.71734)
Supplement: Supplementary file 1 — Data S1. [file ECE3-15-e71734-s001.docx]

**Supplementary material**

**Predicting the phenology of herbivorous insects**

Zimo Yang, Elise Woodruff, David Held, and Nate B Hardy

An article in *Ecology and Evolution*

**Model abbreviations:**

| Abbreviation | Respond variable |
| --- | --- |
| LTE1 | *LT* for egg |
| LTE2 |  |
| LTL1 | *LT* for growth stages |
| LTL2 |  |
| DD1 | *DD* from egg to adult |
| DD2 |  |
| PLTE | *LT* for egg |
| PLTL | *LT* for growth stages |
| PDD | *DD*  from egg to adult |

Models ending with “1” include as a random effect a family-level classification of host plants, whereas those ending in “2“ include nested family- and genus-level host taxa. Models starting with “P” account for shared ancestry among insect species with a phylogenetically-derived variance-covariance matrix, while those without “P” account for shared insect ancestry with nested insect order- and family- level taxa. For further details see Table 2 in main text.

**Table S1. Insect taxonomy coverage of the extended DTR database.** Values in the Cumulative Proportion column accumulate from the top to the bottom; thus the value for Hemiptera is the total proportion accounted for by Lepidoptera + Hemiptera.

| Order | Families | Genera | Species | Records | Proportion | Cumulative Proportion |
| --- | --- | --- | --- | --- | --- | --- |
| Lepidoptera | 25 | 113 | 150 | 234 | 0.389 | 0.389 |
| Hemiptera | 26 | 92 | 112 | 164 | 0.273 | 0.662 |
| Coleoptera | 13 | 67 | 86 | 106 | 0.176 | 0.839 |
| Diptera | 8 | 18 | 41 | 67 | 0.111 | 0.950 |
| Thysanoptera | 2 | 7 | 10 | 12 | 0.020 | 0.970 |
| Orthoptera | 4 | 8 | 8 | 8 | 0.013 | 0.983 |
| Hymenoptera | 3 | 5 | 6 | 7 | 0.012 | 0.995 |
| Psocoptera | 1 | 1 | 2 | 2 | 0.003 | 0.998 |
| Phasmatodea | 1 | 1 | 1 | 1 | 0.002 | 1.000 |
| Summary | 83 | 312 | 416 | 601 | 1.000 | - |

**S2.** **Random intercepts from insect taxonomy in model LTE1.** Levels whose estimate does not include 0 in 95% HDI are marked in bold.

**(a) Insect taxonomy**

| Order | Family | Estimate | 95% HDI | Order | Family | Estimate | 95% HDI |
| --- | --- | --- | --- | --- | --- | --- | --- |
| Coleoptera | Bruchidae | **4.02** | **1.05 ~ 6.92** | Hemiptera (continued) | Miridae | -2.58 | -5.26 ~ 0.32 |
|  | Chrysomelidae | -1.53 | -3.17 ~ 0.09 |  | Pentatomidae | **3.07** | **0.91 ~ 5.38** |
|  | Curculionidae | -0.80 | -2.77 ~ 1.14 |  | Pseudococcidae | 0.71 | -2.51 ~ 4.21 |
|  | Nitidulidae | -0.76 | -4.35 ~ 2.95 |  | Pyrrhocoridae | 1.14 | -2.43 ~ 4.48 |
|  | Scolytidae | 0.50 | -2.11 ~ 3.24 |  | Tingidae | 0.47 | -2.08 ~ 2.99 |
| Diptera | Agromyzidae | -2.63 | -5.28 ~ 0.25 |  | Triozidae | **-2.92** | **-5.57 ~ -0.43** |
|  | Cecidomyiidae | -2.18 | -4.93 ~ 0.61 |  | Tropiduchidae | 1.23 | -2.08 ~ 4.71 |
|  | Chloropidae | -1.30 | -4.74 ~ 2.03 | Lepidoptera | Gelechiidae | 0.65 | -1.57 ~ 2.83 |
|  | Muscidae | 0.96 | -2.40 ~ 4.30 |  | Lasiocampidae | -3.01 | -6.57 ~ 0.43 |
|  | Tephritidae | -1.34 | -3.32 ~ 0.70 |  | Noctuidae | 0.25 | -1.69 ~ 2.11 |
| Hemiptera | Aleyrodidae | 1.47 | -0.95 ~ 3.98 |  | Plutellidae | **-3.65** | **-6.32 ~ -1.03** |
|  | Alydidae | 2.40 | -0.53 ~ 5.29 |  | Pyralidae | 0.94 | -2.33 ~ 4.22 |
|  | Blissidae | 2.72 | -0.68 ~ 5.96 |  | Tortricidae | -0.42 | -3.09 ~ 2.40 |
|  | Cicadellidae | 1.50 | -1.89 ~ 5.07 |  | Yponomeutidae | -0.65 | -4.23 ~ 2.83 |
|  | Coccidae | -0.79 | -3.59 ~ 2.20 | Psocoptera | Liposcelididae | **3.67** | **0.22 ~ 7.30** |
|  | Delphacidae | -0.73 | -2.81 ~ 1.51 | Thysanoptera | Thripidae | -1.69 | -4.10 ~ 0.60 |
|  | Lygaeidae | 1.47 | -1.31 ~ 4.04 | - | - | - | - |

**(b) Diet taxonomy**

| Family | Estimate | 95% HDI | Family | Estimate | 95% HDI |
| --- | --- | --- | --- | --- | --- |
| Fabaceae | 0.72 | -0.54 ~ 2.07 | Malvaceae | 0.68 | -1.18 ~ 2.69 |
| Adoxaceae | -0.02 | -2.22 ~ 1.93 | Moraceae | -0.25 | -2.25 ~ 1.93 |
| Artifical diet | 0.58 | -0.73 ~ 2.16 | Myrtaceae | -0.96 | -2.86 ~ 0.77 |
| Alliaceae | -0.16 | -2.52 ~ 1.99 | Pinaceae | -0.90 | -2.72 ~ 0.75 |
| Amaranthaceae | 0.34 | -1.80 ~ 2.70 | Platanaceae | -0.20 | -2.32 ~ 2.00 |
| Apiaceae | -0.32 | -2.55 ~ 1.90 | Poaceae | 1.54 | -0.05 ~ 3.09 |
| Arecaceae | 0.28 | -1.85 ~ 2.63 | Polygonaceae | -0.19 | -2.35 ~ 1.74 |
| Asteraceae | -1.00 | -3.17 ~ 0.75 | Rosaceae | -0.11 | -1.67 ~ 1.48 |
| Brassicaceae | 0.14 | -1.33 ~ 1.58 | Rubiacea | -0.07 | -2.03 ~ 1.83 |
| Convolvulaceae | -0.30 | -2.38 ~ 1.81 | Salicaceae | -0.13 | -2.06 ~ 1.72 |
| Cucurbitaceae | 0.27 | -1.39 ~ 2.21 | Solanaceae | -0.42 | -1.74 ~ 0.90 |
| Ebanaceae | 0.72 | -1.52 ~ 3.32 | Tamaricaceae | -0.59 | -2.96 ~ 1.28 |
| Ericaceae | -0.40 | -2.57 ~ 1.72 | Ulmaceae | 0.58 | -1.37 ~ 2.89 |

**Table S3. Random intercepts from insect taxonomy in LTE2.** Levels whose estimate does not include 0 in 95% HDI are marked in bold.

**(a) Insect taxonomy**

| Order | Family | Estimate | 95% HDI | Order | Family | Estimate | 95% HDI |
| --- | --- | --- | --- | --- | --- | --- | --- |
| Coleoptera | Bruchidae | **3.88** | **0.65 ~ 6.75** | Hemiptera (continued) | Lygaeidae | 1.74 | -1.13 ~ 4.43 |
|  | Chrysomelidae | -1.45 | -3.08 ~ 0.22 |  | Miridae | -2.09 | -4.92 ~ 0.63 |
|  | Curculionidae | -1.53 | -3.61 ~ 0.40 |  | Pentatomidae | **2.95** | **0.51 ~ 5.37** |
|  | Nitidulidae | -0.85 | -4.44 ~ 2.67 |  | Pseudococcidae | 1.00 | -2.21 ~ 4.35 |
|  | Scolytidae | -0.26 | -2.55 ~ 2.11 |  | Pyrrhocoridae | 1.06 | -2.52 ~ 4.29 |
| Diptera | Agromyzidae | -2.46 | -5.1 ~ 0.25 |  | Tingidae | 0.40 | -2.10 ~ 2.87 |
|  | Cecidomyiidae | -1.71 | -4.45 ~ 0.99 |  | Triozidae | -2.45 | -4.94 ~ 0.12 |
|  | Chloropidae | -0.39 | -3.74 ~ 2.91 |  | Tropiduchidae | 1.03 | -2.39 ~ 4.30 |
|  | Muscidae | 1.12 | -2.30 ~ 4.70 | Lepidoptera | Gelechiidae | 0.22 | -1.95 ~ 2.32 |
|  | Tephritidae | -0.11 | -2.89 ~ 2.62 |  | Lasiocampidae | -3.02 | -6.57 ~ 0.40 |
| Hemiptera | Aleyrodidae | 1.84 | -0.47 ~ 4.31 |  | Noctuidae | 0.14 | -1.92 ~ 2.05 |
|  | Alydidae | 2.22 | -0.55 ~ 5.31 |  | Plutellidae | -3.78 | -6.43 ~ -1.16 |
|  | Blissidae | 2.91 | -0.77 ~ 6.43 |  | Pyralidae | 0.81 | -2.43 ~ 4.21 |
|  | Cicadellidae | 1.49 | -1.89 ~ 4.82 |  | Tortricidae | 0.13 | -3.05 ~ 3.26 |
|  | Coccidae | -0.70 | -3.54 ~ 2.09 |  | Yponomeutidae | -0.88 | -4.38 ~ 2.42 |
|  | Delphacidae | -0.01 | -2.23 ~ 2.25 | Thysanoptera | Thripidae | -1.11 | -3.31 ~ 1.22 |

**(b) Diet taxonomy**

| Family | Genus | Estimate | 95% HDI | Family | Genus | Estimate | 95% HDI |
| --- | --- | --- | --- | --- | --- | --- | --- |
| Adoxaceae | *Viburnum* | -0.03 | -2.30 ~ 1.95 | Moraceae | *Ficus* | -0.33 | -2.65 ~ 1.54 |
| Alliaceae | *Allium* | -0.24 | -2.56 ~ 1.84 | Myrtaceae | *Eucalyptus* | -0.97 | -2.81 ~ 0.79 |
| Amaranthaceae | *Beta* | 0.35 | -1.72 ~ 2.86 | Pinaceae | *Larix* | 0.36 | -1.63 ~ 2.42 |
| Apiaceae | *Daucus* | -0.33 | -2.66 ~ 1.69 |  | *Pinus* | -1.36 | -3.31 ~ 0.38 |
| Arecaceae | *Phoenix* | 0.25 | -2.07 ~ 2.42 | Platanaceae | *Platanus* | -0.20 | -2.36 ~ 1.92 |
| Asteraceae | *Chrysanthemum* | -0.16 | -2.22 ~ 1.97 | Poaceae | *Hymenachne* | 0.73 | -1.34 ~ 3.38 |
|  | *Parthenium* | -1.28 | -4.01 ~ 0.83 |  | *Lolium* | -0.10 | -2.35 ~ 2.01 |
| Brassicaceae | *Brassica* | 0.13 | -1.29 ~ 1.67 |  | *Oryza* | 0.63 | -0.94 ~ 2.42 |
|  | *Lepidium* | -0.22 | -2.49 ~ 1.76 |  | *Sorghum* | 0.29 | -1.88 ~ 2.60 |
| Convolvulaceae | *Ipomoea* | -0.26 | -2.32 ~ 1.64 |  | *Triticum* | 0.18 | -1.76 ~ 2.15 |
| Cucurbitaceae | *Cucumis* | 0.30 | -1.46 ~ 2.02 |  | *Zea* | 0.94 | -0.68 ~ 3.12 |
| Ebanaceae | *Diospyros* | 0.66 | -1.43 ~ 3.05 | Polygonaceae | *Rumex* | -0.23 | -2.39 ~ 1.58 |
| Ericaceae | *Rhododendron* | -0.29 | -2.46 ~ 1.84 | Rosaceae | *Malus* | -0.71 | -2.72 ~ 0.98 |
| Fabaceae | *Arachis* | -0.06 | -1.62 ~ 1.51 |  | *Prunus* | 0.36 | -1.66 ~ 2.47 |
|  | *Glycine* | 0.39 | -1.33 ~ 2.08 | Rubiacea | *Coffea* | -0.17 | -2.10 ~ 1.68 |
|  | *Medicago* | 0.29 | -1.70 ~ 2.32 | Salicaceae | *Populus* | 0.59 | -1.45 ~ 2.93 |
|  | *Phaseolus* | 0.22 | -1.70 ~ 2.05 |  | *Salix* | -0.81 | -3.25 ~ 1.24 |
|  | *Vicia* | 0.06 | -2.05 ~ 2.14 | Solanaceae | *Capsicum* | 0.47 | -1.23 ~ 2.46 |
|  | *Vigna* | 0.55 | -1.02 ~ 2.49 |  | *Solanum* | -0.74 | -2.2 ~ 0.54 |
| Malvaceae | *Abelmoschus* | 0.26 | -1.93 ~ 2.5 | Tamaricaceae | *Tamarix* | -0.67 | -3.10 ~ 1.26 |
|  | *Corchorus* | 0.32 | -1.59 ~ 2.45 | Ulmaceae | *Ulmus* | 0.49 | -1.39 ~ 2.7 |
|  | *Hibiscus* | 0.27 | -2.01 ~ 2.50 | - | - | - | - |

**Table S4. Random intercepts from insect taxonomy in LTL1.** Levels whose estimate does not include 0 in 95% HDI are marked in bold.

**(a) Insect taxonomy**

| Order | Family | Estimate | 95% HDI | Order | Family | Estimate | 95% HDI |
| --- | --- | --- | --- | --- | --- | --- | --- |
| Coleoptera | Bruchidae | 0.68 | -2.53 ~ 4.05 | Hemiptera (continued) | Delphacidae | 0.09 | -2.10 ~ 2.30 |
|  | Cerambycidae | 2.00 | -1.49 ~ 5.33 |  | Diaspididae | -0.72 | -4.69 ~ 3.28 |
|  | Chrysomelidae | -1.22 | -3.16 ~ 0.65 |  | Fulgoridae | 0.28 | -3.64 ~ 4.44 |
|  | Curculionidae | 0.19 | -1.95 ~ 2.29 |  | Lygaeidae | **3.10** | **0.01 ~ 6.47** |
|  | Nitidulidae | 0.41 | -3.96 ~ 4.87 |  | Miridae | -2.26 | -6.23 ~ 1.42 |
|  | Scolytidae | 0.79 | -1.96 ~ 3.47 |  | Pentatomidae | **3.05** | **0.24 ~ 5.82** |
|  | Tenebrionidae | 0.65 | -4.17 ~ 5.34 |  | Pseudococcidae | 0.49 | -2.62 ~ 3.62 |
| Diptera | Agromyzidae | -2.29 | -5.51 ~ 0.83 |  | Pyrrhocoridae | 0.53 | -3.25 ~ 4.51 |
|  | Cecidomyiidae | -5.54 | -8.97 ~ -2.20 |  | Tingidae | 1.84 | -0.97 ~ 4.54 |
|  | Chloropidae | 0.65 | -3.05 ~ 4.67 |  | Triozidae | **-3.85** | **-6.59 ~ -0.94** |
|  | Muscidae | -0.99 | -4.90 ~ 3.07 |  | Tropiduchidae | 0.83 | -3.14 ~ 4.86 |
|  | Tephritidae | -1.06 | -3.26 ~ 1.00 | Lepidoptera | Gelechiidae | -0.23 | -2.48 ~ 2.09 |
| Hemiptera | Adelgidae | **-6.60** | **-11.08 ~ -1.91** |  | Noctuidae | -0.40 | -2.53 ~ 1.80 |
|  | Aleyrodidae | 1.53 | -1.14 ~ 4.44 |  | Plutellidae | **-4.38** | **-7.22 ~ -1.34** |
|  | Alydidae | 2.99 | -0.31 ~ 6.31 |  | Pyralidae | -0.22 | -4.12 ~ 3.81 |
|  | Aphididae | **-4.85** | **-6.81 ~ -2.64** |  | Tortricidae | 1.53 | -1.54 ~ 4.77 |
|  | Blissidae | 1.87 | -2.11 ~ 5.75 |  | Yponomeutidae | -1.93 | -6.19 ~ 2.06 |
|  | Cercopidae | -0.78 | -5.09 ~ 3.73 | Orthoptera | Acaridae | **4.24** | **0.03 ~ 8.52** |
|  | Cicadellidae | 2.88 | -1.26 ~ 6.75 | Psocoptera | Liposcelididae | **5.81** | **1.78 ~ 10.11** |
|  | Coccidae | 0.64 | -3.27 ~ 4.35 | Thysanoptera | Thripidae | -0.46 | -3.06 ~ 2.20 |

**(b) Diet taxonomy**

| Family | Estimate | 95% HDI | Family | Estimate | 95% HDI |
| --- | --- | --- | --- | --- | --- |
| Fabaceae | 0.06 | -0.77 ~ 0.92 | Myrtaceae | -0.21 | -1.49 ~ 0.68 |
| Adoxaceae | -0.03 | -1.11 ~ 1.06 | Pinaceae | -0.01 | -1.01 ~ 0.95 |
| Artificial diet | 0.28 | -0.48 ~ 1.44 | Platanaceae | -0.09 | -1.26 ~ 0.92 |
| Alliaceae | -0.05 | -1.31 ~ 0.96 | Poaceae | 0.00 | -0.83 ~ 0.81 |
| Amaranthaceae | -0.03 | -1.10 ~ 1.05 | Polygonaceae | -0.06 | -1.24 ~ 1.00 |
| Apiaceae | -0.01 | -1.21 ~ 1.03 | Rosaceae | 0.01 | -0.90 ~ 0.90 |
| Arecaceae | 0.03 | -1.14 ~ 1.16 | Rubiacea | -0.07 | -1.17 ~ 1.00 |
| Asteraceae | 0.01 | -1.12 ~ 1.01 | Rutaceae | -0.12 | -1.35 ~ 0.89 |
| Brassicaceae | -0.01 | -0.87 ~ 0.95 | Salicaceae | 0.01 | -0.97 ~ 1.14 |
| Celestraceae | -0.02 | -1.15 ~ 1.16 | Simaroubaceae | 0.01 | -1.12 ~ 1.19 |
| Cucurbitaceae | 0.20 | -0.73 ~ 1.46 | Solanaceae | 0.03 | -0.79 ~ 1.04 |
| Ericaceae | 0.03 | -1.04 ~ 1.25 | Ulmaceae | 0.08 | -0.95 ~ 1.29 |
| Malvaceae | 0.20 | -0.79 ~ 1.41 | Violaceae | -0.13 | -1.32 ~ 0.93 |
| Moraceae | -0.10 | -1.27 ~ 0.97 | - | - | - |

**Table S5. Random intercepts from insect taxonomy in LTL2.** Levels whose estimate does not include 0 in 95% HDI are marked in bold.

**(a) Insect taxonomy**

| Order | Family | Estimate | 95% HDI | Order | Family | Estimate | 95% HDI |
| --- | --- | --- | --- | --- | --- | --- | --- |
| Coleoptera | Bruchidae | 0.75 | -2.35 ~ 4.07 | Hemiptera (continued) | Delphacidae | 0.58 | -1.63 ~ 2.92 |
|  | Cerambycidae | 3.03 | -0.84 ~ 7.03 |  | Diaspididae | -0.47 | -4.23 ~ 3.38 |
|  | Chrysomelidae | -0.78 | -2.60 ~ 1.04 |  | Fulgoridae | 0.57 | -3.39 ~ 4.45 |
|  | Curculionidae | -0.47 | -2.77 ~ 1.68 |  | Lygaeidae | **3.55** | **0.78 ~ 6.77** |
|  | Nitidulidae | 0.46 | -3.75 ~ 4.78 |  | Miridae | -2.04 | -5.64 ~ 1.88 |
|  | Scolytidae | 0.96 | -1.58 ~ 3.62 |  | Pentatomidae | **3.58** | **0.97 ~ 6.19** |
| Diptera | Agromyzidae | -2.29 | -5.23 ~ 0.70 |  | Pseudococcidae | 0.76 | -2.20 ~ 3.86 |
|  | Cecidomyiidae | **-5.37** | **-8.56 ~ -1.90** |  | Pyrrhocoridae | 1.08 | -2.52 ~ 5.00 |
|  | Chloropidae | 0.75 | -2.79 ~ 4.78 |  | Tingidae | 2.17 | -0.38 ~ 4.88 |
|  | Muscidae | -0.66 | -4.75 ~ 2.95 |  | Triozidae | **-3.69** | **-6.57 ~ -0.95** |
|  | Tephritidae | -2.79 | -5.91 ~ 0.21 |  | Tropiduchidae | 1.37 | -2.55 ~ 5.28 |
| Hemiptera | Adelgidae | **-6.34** | **-10.72 ~ -1.90** | Lepidoptera | Gelechiidae | 0.04 | -2.16 ~ 2.27 |
|  | Aleyrodidae | 1.68 | -0.96 ~ 4.37 |  | Noctuidae | 0.29 | -1.90 ~ 2.56 |
|  | Alydidae | **3.74** | **0.55 ~ 7.11** |  | Plutellidae | **-4.17** | **-7.02 ~ -1.45** |
|  | Aphididae | **-4.45** | **-6.59 ~ -2.46** |  | Pyralidae | 0.12 | -3.64 ~ 4.06 |
|  | Blissidae | 2.37 | -1.13 ~ 6.43 |  | Tortricidae | **3.77** | **0.03 ~ 7.67** |
|  | Cercopidae | -0.74 | -5.34 ~ 3.47 |  | Yponomeutidae | -1.81 | -5.62 ~ 2.06 |
|  | Cicadellidae | 3.48 | -0.57 ~ 7.04 | Thysanoptera | Thripidae | -0.33 | -2.84 ~ 2.11 |
|  | Coccidae | 0.90 | -2.75 ~ 4.65 | - | - | - | - |

**(b) Diet taxonomy**

| Family | Genus | Estimate | 95% HDI | Family | Genus | Estimate | 95% HDI |
| --- | --- | --- | --- | --- | --- | --- | --- |
| Adoxaceae | *Viburnum* | -0.04 | -1.47 ~ 1.28 | Pinaceae | *Larix* | 0.22 | -1.14 ~ 1.81 |
| Alliaceae | *Allium* | -0.09 | -1.70 ~ 1.26 |  | *Pinus* | 0.00 | -1.31 ~ 1.16 |
| Amaranthaceae | *Beta* | -0.06 | -1.54 ~ 1.23 |  | *Tsuga* | -0.33 | -2.24 ~ 1.07 |
| Apiaceae | *Daucus* | 0.00 | -1.59 ~ 1.47 | Platanaceae | *Platanus* | -0.13 | -1.64 ~ 1.29 |
| Arecaceae | *Phoenix* | 0.08 | -1.42 ~ 1.66 | Poaceae | *Hordeum* | 0.01 | -1.17 ~ 1.18 |
| Asteraceae | *Chrysanthemum* | 0.05 | -1.48 ~ 1.43 |  | *Hymenachne* | 0.12 | -1.23 ~ 1.85 |
| Brassicaceae | *Brassica* | 0.12 | -0.86 ~ 1.28 |  | *Lolium* | 0.04 | -1.59 ~ 1.47 |
|  | *Lepidium* | -0.28 | -2.11 ~ 1.00 |  | *Oryza* | 0.05 | -1.16 ~ 1.32 |
| Celestraceae | *Euonymus* | -0.02 | -1.48 ~ 1.42 |  | *Sorghum* | -0.03 | -1.49 ~ 1.48 |
| Cucurbitaceae | *Cucumis* | 0.37 | -0.75 ~ 1.97 |  | *Triticum* | -0.28 | -1.94 ~ 1.00 |
| Ericaceae | *Rhododendron* | 0.05 | -1.28 ~ 1.56 |  | *Zea* | 0.35 | -0.84 ~ 1.98 |
| Fabaceae | *Arachis* | 0.26 | -0.83 ~ 1.73 | Polygonaceae | *Rumex* | -0.12 | -1.76 ~ 1.09 |
|  | *Glycine* | -0.12 | -1.38 ~ 1.04 | Rosaceae | *Malus* | -0.31 | -1.77 ~ 0.76 |
|  | *Medicago* | 0.14 | -1.22 ~ 1.47 |  | *Prunus* | 0.23 | -1.10 ~ 1.73 |
|  | *Phaseolus* | -0.01 | -1.38 ~ 1.33 | Rubiacea | *Coffea* | -0.02 | -1.42 ~ 1.28 |
|  | *Vicia* | -0.33 | -2.06 ~ 0.98 | Rutaceae | *Citrus* | -0.25 | -2.07 ~ 1.06 |
|  | *Vigna* | 0.17 | -1.05 ~ 1.53 | Salicaceae | *Populus* | 0.22 | -1.02 ~ 1.89 |
| Malvaceae | *Abelmoschus* | 0.06 | -1.52 ~ 1.49 |  | *Salix* | -0.22 | -1.93 ~ 1.07 |
|  | *Corchorus* | 0.04 | -1.44 ~ 1.44 | Simaroubaceae | *Ailanthus* | 0.02 | -1.45 ~ 1.51 |
|  | *Gossypium* | 0.09 | -1.24 ~ 1.55 | Solanaceae | *Capsicum* | 0.11 | -1.11 ~ 1.55 |
|  | *Hibiscus* | 0.28 | -0.95 ~ 2.11 |  | *Solanum* | 0.11 | -0.95 ~ 1.26 |
| Moraceae | *Ficus* | -0.18 | -1.77 ~ 1.09 | Ulmaceae | *Ulmus* | 0.17 | -1.17 ~ 1.74 |
| Myrtaceae | *Eucalyptus* | -0.32 | -1.81 ~ 0.75 | Violaceae | *Viola* | -0.27 | -2.11 ~ 0.88 |

**Table S6. Random intercepts from insect taxonomy in DD1.** Levels whose estimate does not include 0 in 95% HDI are marked in bold.

**(a) Insect taxonomy**

| Order | Family | Estimate | 95% HDI | Order | Family | Estimate | 95% HDI |
| --- | --- | --- | --- | --- | --- | --- | --- |
| Coleoptera | Bruchidae | **0.40** | **0.00 ~ 0.81** | Hemiptera (continued) | Lygaeidae | 0.08 | -0.36 ~ 0.56 |
|  | Buprestidae | **-0.85** | **-1.53 ~ -0.16** |  | Miridae | 0.10 | -0.42 ~ 0.65 |
|  | Chrysomelidae | 0.09 | -0.17 ~ 0.34 |  | Pentatomidae | 0.30 | -0.05 ~ 0.69 |
|  | Curculionidae | 0.12 | -0.18 ~ 0.44 |  | Pseudococcidae | -0.02 | -0.49 ~ 0.44 |
|  | Scolytidae | -0.01 | -0.45 ~ 0.43 |  | Psyllidae | -0.28 | -0.84 ~ 0.28 |
|  | Tenebrionidae | 0.32 | -0.22 ~ 0.88 |  | Pyrrhocoridae | -0.07 | -0.61 ~ 0.49 |
| Diptera | Agromyzidae | 0.04 | -0.39 ~ 0.46 |  | Tingidae | 0.05 | -0.37 ~ 0.48 |
|  | Cecidomyiidae | 0.14 | -0.25 ~ 0.55 |  | Triozidae | -0.06 | -0.47 ~ 0.36 |
|  | Chloropidae | 0.28 | -0.28 ~ 0.82 |  | Tropiduchidae | 0.41 | -0.18 ~ 1.02 |
|  | Muscidae | 0.05 | -0.49 ~ 0.57 | Hymenoptera | Cynipidae | -0.14 | -0.68 ~ 0.42 |
|  | Tephritidae | 0.01 | -0.27 ~ 0.31 | Lepidoptera | Gelechiidae | -0.14 | -0.47 ~ 0.19 |
| Hemiptera | Aleyrodidae | 0.05 | -0.31 ~ 0.41 |  | Noctuidae | 0.00 | -0.34 ~ 0.34 |
|  | Alydidae | -0.29 | -0.79 ~ 0.18 |  | Plutellidae | -0.43 | -0.89 ~ 0.01 |
|  | Aphididae | **-0.71** | **-1.00 ~ -0.42** |  | Pyralidae | -0.15 | -0.48 ~ 0.15 |
|  | Blissidae | 0.17 | -0.35 ~ 0.68 |  | Tortricidae | 0.12 | -0.27 ~ 0.52 |
|  | Cicadellidae | -0.29 | -0.78 ~ 0.19 |  | Yponomeutidae | 0.18 | -0.40 ~ 0.76 |
|  | Coccidae | 0.33 | -0.18 ~ 0.91 |  | Zygaenidae | -0.02 | -0.59 ~ 0.49 |
|  | Delphacidae | -0.10 | -0.44 ~ 0.24 | Psocoptera | Liposcelididae | -0.10 | -0.63 ~ 0.46 |
|  | Diaspididae | 0.42 | -0.08 ~ 0.88 | Thysanoptera | Phlaeothripidae | -0.09 | -0.65 ~ 0.45 |
|  | Fulgoridae | 0.24 | -0.31 ~ 0.82 |  | Thripidae | -0.20 | -0.57 ~ 0.16 |

**(b) Diet taxonomy**

| Family | Estimate | 95% HDI | Family | Estimate | 95% HDI |
| --- | --- | --- | --- | --- | --- |
| Fabaceae | -0.05 | -0.29 ~ 0.16 | Malvaceae | -0.09 | -0.43 ~ 0.20 |
| Adoxaceae | 0.01 | -0.36 ~ 0.40 | Moraceae | 0.05 | -0.29 ~ 0.41 |
| Artificial diet | -0.10 | -0.34 ~ 0.11 | Myrtaceae | 0.19 | -0.13 ~ 0.63 |
| Alliaceae | 0.06 | -0.30 ~ 0.54 | Pinaceae | 0.15 | -0.11 ~ 0.46 |
| Amaranthaceae | 0.06 | -0.26 ~ 0.44 | Platanaceae | -0.01 | -0.42 ~ 0.37 |
| Anacardiaceae | -0.05 | -0.44 ~ 0.32 | Poaceae | -0.12 | -0.33 ~ 0.06 |
| Apiaceae | 0.12 | -0.28 ~ 0.61 | Polygonaceae | -0.08 | -0.51 ~ 0.25 |
| Arecaceae | 0.14 | -0.26 ~ 0.62 | Rosaceae | -0.06 | -0.33 ~ 0.16 |
| Asteraceae | 0.04 | -0.28 ~ 0.38 | Rubiacea | 0.14 | -0.19 ~ 0.56 |
| Betulaceae | -0.29 | -0.94 ~ 0.16 | Rutaceae | -0.13 | -0.49 ~ 0.16 |
| Brassicaceae | -0.14 | -0.41 ~ 0.09 | Salicaceae | -0.01 | -0.34 ~ 0.34 |
| Convolvulaceae | 0.01 | -0.34 ~ 0.43 | Solanaceae | 0.10 | -0.09 ~ 0.31 |
| Cucurbitaceae | -0.14 | -0.47 ~ 0.12 | Tamaricaceae | -0.01 | -0.40 ~ 0.35 |
| Ericaceae | 0.16 | -0.21 ~ 0.67 | Ulmaceae | -0.02 | -0.42 ~ 0.33 |
| Fagaceae | -0.05 | -0.53 ~ 0.32 | Vitaceae | -0.01 | -0.34 ~ 0.35 |
| Juglandaceae | 0.15 | -0.16 ~ 0.54 | - | - | - |

**Table S7.** **Random intercept from insect taxonomy in DD2.** Levels whose estimate does not include 0 in 95% HDI are marked in bold.

**(a) Insect taxonomy**

| Order | Family | Estimate | 95% HDI | Order | Family | Estimate | 95% HDI |
| --- | --- | --- | --- | --- | --- | --- | --- |
| Coleoptera | Bruchidae | 0.35 | -0.08 ~ 0.77 | Hemiptera (continued) | Lygaeidae | 0.03 | -0.42 ~ 0.49 |
|  | Buprestidae | -0.70 | -1.39 ~ -0.04 |  | Miridae | 0.04 | -0.47 ~ 0.56 |
|  | Chrysomelidae | 0.12 | -0.14 ~ 0.37 |  | Pentatomidae | 0.32 | -0.07 ~ 0.73 |
|  | Curculionidae | 0.19 | -0.17 ~ 0.54 |  | Pseudococcidae | -0.06 | -0.53 ~ 0.40 |
|  | Scolytidae | 0.07 | -0.32 ~ 0.49 |  | Psyllidae | -0.21 | -0.74 ~ 0.32 |
| Diptera | Agromyzidae | 0.05 | -0.38 ~ 0.47 |  | Pyrrhocoridae | -0.06 | -0.61 ~ 0.47 |
|  | Cecidomyiidae | 0.15 | -0.26 ~ 0.54 |  | Tingidae | 0.09 | -0.34 ~ 0.49 |
|  | Chloropidae | 0.17 | -0.35 ~ 0.77 |  | Triozidae | -0.09 | -0.48 ~ 0.32 |
|  | Muscidae | 0.02 | -0.54 ~ 0.58 |  | Tropiduchidae | 0.38 | -0.22 ~ 0.94 |
|  | Tephritidae | 0.17 | -0.22 ~ 0.55 | Hymenoptera | Cynipidae | -0.10 | -0.64 ~ 0.45 |
| Hemiptera | Aleyrodidae | 0.08 | -0.30 ~ 0.42 | Lepidoptera | Gelechiidae | -0.14 | -0.47 ~ 0.18 |
|  | Alydidae | -0.23 | -0.73 ~ 0.27 |  | Noctuidae | 0.01 | -0.35 ~ 0.37 |
|  | Aphididae | -0.60 | -0.94 ~ -0.28 |  | Plutellidae | -0.31 | -0.79 ~ 0.10 |
|  | Blissidae | 0.11 | -0.41 ~ 0.65 |  | Pyralidae | -0.14 | -0.48 ~ 0.18 |
|  | Cicadellidae | -0.27 | -0.75 ~ 0.16 |  | Tortricidae | 0.05 | -0.36 ~ 0.50 |
|  | Coccidae | 0.29 | -0.21 ~ 0.84 |  | Yponomeutidae | 0.16 | -0.40 ~ 0.73 |
|  | Delphacidae | -0.24 | -0.64 ~ 0.12 |  | Zygaenidae | -0.01 | -0.53 ~ 0.51 |
|  | Diaspididae | 0.36 | -0.09 ~ 0.85 | Thysanoptera | Phlaeothripidae | -0.08 | -0.61 ~ 0.45 |
|  | Fulgoridae | 0.24 | -0.30 ~ 0.80 |  | Thripidae | -0.20 | -0.58 ~ 0.17 |

**(b) Diet taxonomy**

| Family | Genus | Estimate | 95% HDI | Family | Genus | Estimate | 95% HDI |
| --- | --- | --- | --- | --- | --- | --- | --- |
| Adoxaceae | *Viburnum* | 0.03 | -0.40 ~ 0.45 | Malvaceae (continued) | *Gossypium* | -0.14 | -0.62 ~ 0.29 |
| Alliaceae | *Allium* | 0.09 | -0.34 ~ 0.58 |  | *Hibiscus* | -0.04 | -0.49 ~ 0.40 |
| Amaranthaceae | *Beta* | 0.09 | -0.30 ~ 0.50 | Moraceae | *Ficus* | 0.07 | -0.32 ~ 0.47 |
| Anacardiaceae | *Pistacia* | -0.05 | -0.48 ~ 0.36 | Myrtaceae | *Eucalyptus* | 0.26 | -0.13 ~ 0.69 |
| Apiaceae | *Daucus* | 0.14 | -0.30 ~ 0.66 | Pinaceae | *Larix* | -0.16 | -0.60 ~ 0.24 |
| Arecaceae | *Phoenix* | 0.21 | -0.23 ~ 0.77 |  | *Pinus* | 0.28 | -0.07 ~ 0.59 |
| Asteraceae | *Chrysanthemum* | -0.07 | -0.51 ~ 0.34 | Platanaceae | *Platanus* | -0.02 | -0.47 ~ 0.41 |
|  | *Parthenium* | 0.15 | -0.26 ~ 0.60 | Poaceae | *Hordeum* | -0.31 | -0.68 ~ 0.02 |
| Betulaceae | *Betula* | -0.39 | -0.99 ~ 0.12 |  | *Hymenachne* | 0.07 | -0.38 ~ 0.52 |
| Brassicaceae | *Brassica* | -0.22 | -0.51 ~ 0.04 |  | *Lolium* | 0.09 | -0.32 ~ 0.57 |
|  | *Lepidium* | 0.14 | -0.25 ~ 0.62 |  | *Oryza* | 0.11 | -0.19 ~ 0.41 |
| Convolvulaceae | *Ipomoea* | 0.02 | -0.39 ~ 0.45 |  | *Sorghum* | 0.01 | -0.43 ~ 0.46 |
| Cucurbitaceae | *Cucumis* | -0.22 | -0.56 ~ 0.10 |  | *Triticum* | -0.15 | -0.51 ~ 0.16 |
|  | *Cucurbita* | 0.01 | -0.43 ~ 0.44 |  | *Zea* | -0.15 | -0.53 ~ 0.20 |
| Ericaceae | *Rhododendron* | 0.21 | -0.21 ~ 0.73 | Polygonaceae | *Rumex* | -0.11 | -0.56 ~ 0.30 |
| Fabaceae | *Arachis* | 0.02 | -0.29 ~ 0.33 | Rosaceae | *Malus* | 0.00 | -0.33 ~ 0.36 |
|  | *Cicer* | 0.02 | -0.40 ~ 0.45 |  | *Prunus* | -0.08 | -0.40 ~ 0.23 |
|  | *Glycine* | -0.04 | -0.42 ~ 0.33 | Rubiacea | *Coffea* | 0.20 | -0.20 ~ 0.63 |
|  | *Medicago* | -0.15 | -0.56 ~ 0.22 |  | *Citrus* | -0.20 | -0.57 ~ 0.14 |
|  | *Phaseolus* | 0.11 | -0.24 ~ 0.49 | Salicaceae | *Populus* | -0.13 | -0.56 ~ 0.30 |
|  | *Vicia* | -0.02 | -0.45 ~ 0.43 |  | *Salix* | 0.10 | -0.32 ~ 0.53 |
|  | *Vigna* | 0.00 | -0.33 ~ 0.35 | Solanaceae | *Capsicum* | -0.10 | -0.49 ~ 0.26 |
| Fagaceae | *Castanea* | -0.06 | -0.52 ~ 0.38 |  | *Solanum* | 0.17 | -0.05 ~ 0.40 |
| Juglandaceae | *Julgans* | 0.19 | -0.19 ~ 0.60 | Tamaricaceae | *Tamarix* | 0.00 | -0.43 ~ 0.41 |
| Malvaceae | *Abelmoschus* | -0.03 | -0.47 ~ 0.44 | Ulmaceae | *Ulmus* | -0.02 | -0.43 ~ 0.41 |
|  | *Corchorus* | 0.02 | -0.41 ~ 0.45 | Vitaceae | *Vitis* | 0.07 | -0.31 ~ 0.47 |

**Table S8. Variance explained by random variables in models of *LT* and *DD*.**

| LTE1 | | | | LTE2 | | | |
| --- | --- | --- | --- | --- | --- | --- | --- |
| Group | Value | | *SE* | Group | Value | *SE* | |
| Insect order:family | 2.49 | | 0.49 | Insect order:family | 2.35 | 0.49 | |
| Diet family | 1.10 | | 0.46 | Diet family: genus | 1.07 | 0.47 | |
| Sigma | 2.08 | | 0.18 | Sigma | 2.02 | 0.22 | |
| LTL1 | | | | LTL2 | | | |
| Group | Value | *SE* | | Group | Value | | *SE* |
| Insect order:family | 3.14 | 0.54 | | Insect order:family | 3.11 | | 0.55 |
| Diet family | 0.43 | 0.33 | | Diet family: genus | 0.58 | | 0.42 |
| Sigma | 2.42 | 0.19 | | Sigma | 2.20 | | 0.21 |
| DD1 | | | | DD2 | | | |
| Group | Value | | *SE* | Group | Value | *SE* | |
| Insect order:family | 0.36 | | 0.08 | Insect order:family | 0.34 | 0.08 | |
| Diet family | 0.19 | | 0.11 | Diet family: genus | 0.25 | 0.08 | |
| Sigma | 0.38 | | 0.03 | Sigma | 0.38 | 0.03 | |
| PLTE | | | | PLTL | | | |
| Group | Value | | *SD* | Group | Value | *SD* | |
| Insect species | 1.19 | | 1.09 | Insect species | 2.08 | 1.44 | |
| Insect phylogeny | 3.74 | | 1.93 | Insect phylogeny | 1.78 | 1.33 | |
| Diet family | 1.63 | | 1.28 | Diet family | 0.30 | 0.55 | |
| - | - | | - | Measurement type | 15.58 | 3.95 | |
| Sigma | 0.54 | | 0.73 | Sigma | 0.40 | 0.64 | |
| PDD | | | | | | | |
| Group | | | Value | | *SD* | | |
| Insect species | | | 0.01 | | 0.08 | | |
| Insect phylogeny | | | 0.03 | | 0.16 | | |
| Diet family | | | <0.01 | | 0.07 | | |
| Measurement type | | | 0.11 | | 0.34 | | |
| Sigma | | | 0.01 | | 0.11 | | |

**Figure S1**


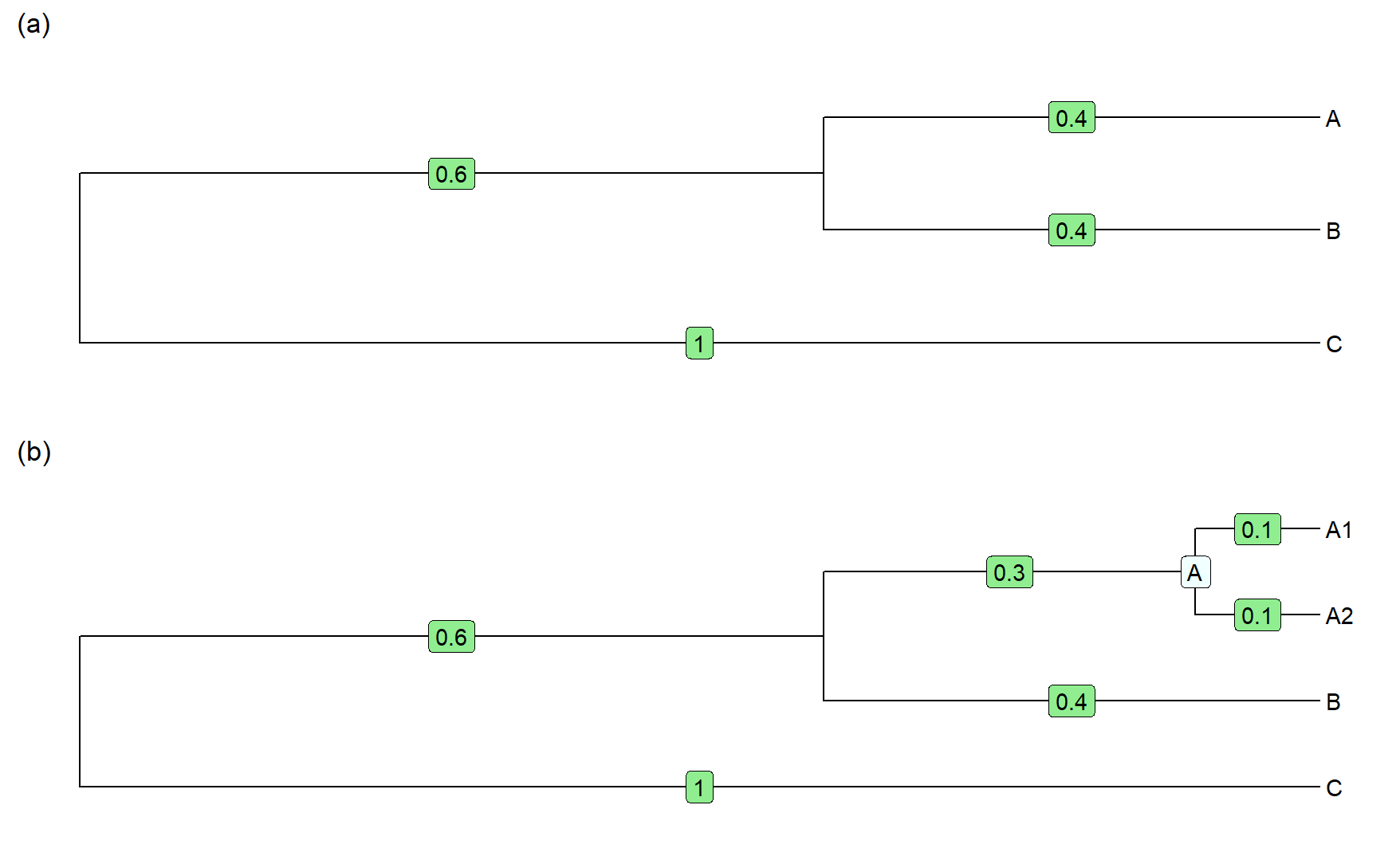


**Figure S1. Incorporation of multiple conspecific populations into phylogenetic models. (a) A hypothetical phylogenetic tree.** The 3 tips of this tree are species A, B, and C. The evolutionary length from root to any tip is the same, so the tree is ultrametric. The length of each branch is shown in a green box. **(b) The modified tree.** Suppose we have two phenology models for species A. To incorporate these data in our phylogenetic comparative models, we make species A an internal node that subtends two “pseudo tips, ” A1 and A2, of a small length *L*. Here, for clarity, we use a value for *L* of 0.1, but in our analysis we used a smaller value, 1e-6. To keep the tree ultrametric we subtract *L* from the length of the branch subtending internal A. We then use this modified tree for the calculation of the variance-covariance matrix used as a random effect in the phylogenetic models.

**Figure S2**


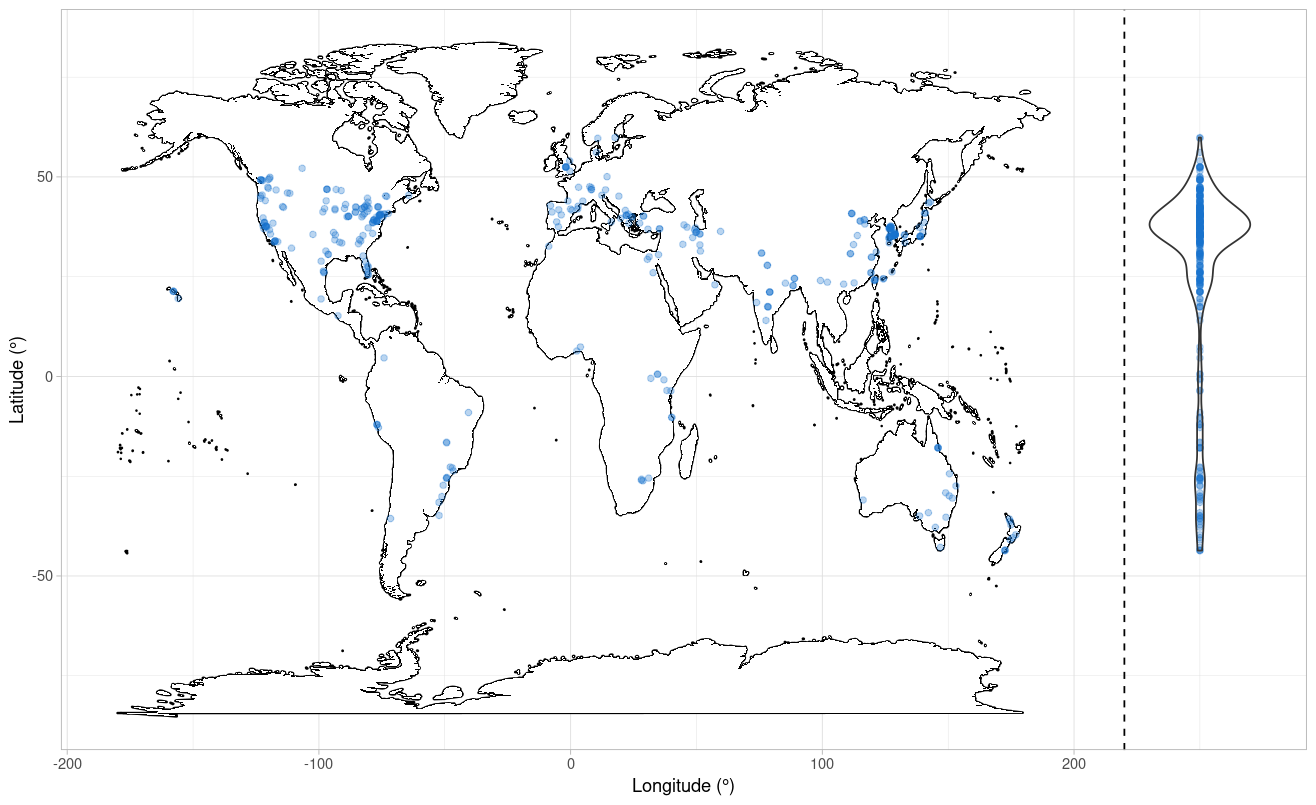


**Figure S2.** **Distribution of source populations with latitudinal range <10 degrees.** Increased opacity indicates overlapping source populations. The violin plot on the right is an alignment of all points by latitudes. Not all the studies plotted here were analyzed due to missing data for other parameters, but the distribution patterns are similar for the data subsets used for each model. The map is based on the ‘map’ data of R package *maps* (Becker et al. 2024).


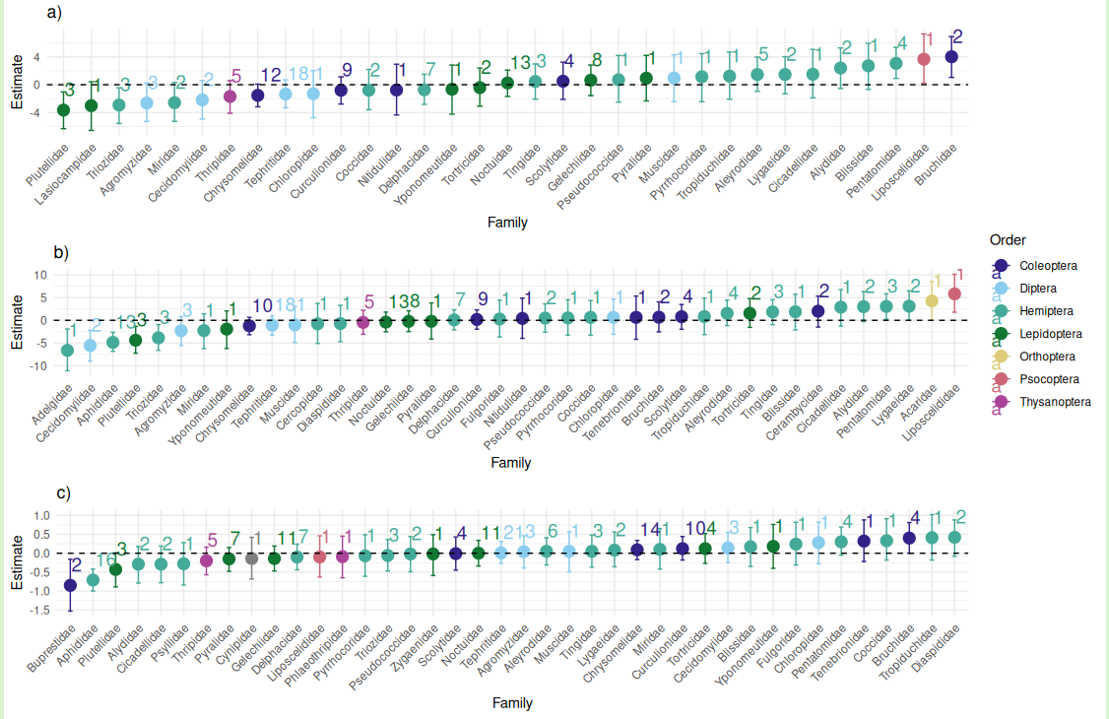


**Figure S3.** **Level-specific posterior distribution of insect order-family random effect estimates of a) LTE1, b) LTL1, c) DD1.** Points represent distribution mean and error bars represent 95% CI. The numbers are a count of phenology model parametrizations in each family. Although in each model the order and family random effects explain a large portion of variance in the response variable (see Table S8), most level-specific estimates are ambiguous because they have large 95% CIs that cross zero. Other models are qualitatively similar.

**REFERENCES**

Becker, R. A., A. R. Wilks, R. Brownrigg, T. P. Minka, and A. Deckmyn. 2024, November 10. maps: Draw Geographical Maps.
